# Effect of Anemia on Readmission and Death in Octogenarian Patients with Lower Respiratory Tract Infections: A Retrospective Cohort Study

**DOI:** 10.1155/2022/4566936

**Published:** 2022-10-27

**Authors:** Yu He, Ying Chen, Kai Cao, Hui Zheng

**Affiliations:** ^1^Department of Geriatrics, Beijing Tongren Hospital, Capital Medical University, Beijing, China; ^2^Beijing Institute of Ophthalmology, Beijing Tongren Hospital, Capital Medical University, Beijing, China

## Abstract

**Objectives:**

Lower respiratory tract infection (LRTI) in the octogenarian population is a highly prevalent disorder associated with increased mortality rates. Anemia is a common disorder in older adults and is often left untreated. We investigated whether anemia was a risk factor for LRTI-caused readmission and death in octogenarian patients.

**Design:**

A retrospective cohort study was designed. *Participants*. Old patients (age: ≥80 years) hospitalized at the Department of Geriatrics to undergo treatment for LRTIs were included. A total of 215 patients (mean age: 88.73 years; 77.2% men) were studied. The patients were divided into two groups (anemia and non-anemia) based on the hemoglobin level. They were followed up for 1 year after discharge or until mortality event. The primary follow-up outcome was LRTI-caused readmission and/or death.

**Results:**

The risk of readmission due to LTRI within 1 year of discharge was 2.308 times higher in the anemia group than the risk for the non-anemia group. The number of patients experiencing ≥2 readmissions in the anemia group was significantly higher than that in the non-anemia group (35 (23.5%) vs. 2 (3%), *P* < 0.001). To prevent readmission of one case, only 2.65 cases with anemia needed to be treated. Furthermore, the risk of LRTI-related deaths was 6.644 times higher in the anemia group than the risk for the non-anemia group. To prevent death of one case, only 3.9 cases with anemia needed to be treated. Statistic results revealed that hemoglobin was an independent protective factor for LRTI-caused readmission (logistic regression method, OR, 0.969; 95% CI, 0.950–0.989) and deaths (Cox regression method, *β*, −0.037, HR, 0.964; 95% CI, 0.934–0.994).

**Conclusions:**

Anemia is a widely prevalent and relevant risk factor associated with LRTI-caused readmission and death within 1 year of discharge in octogenarian patients. *Trial Registration*. This trial is registered with NCT05355324.

## 1. Background

Lower respiratory tract infection (LRTI) is used as a synonym for pneumonia or bronchiolitis, as reported by the Global Burden of Diseases, Injuries, and Risk Factors Study (GBD) [1]. LRTI poses a severe threat to the health of older adults worldwide [2–5]. It has been reported that LRTI is the 5th leading cause of death in adults ≥70 years of age [6]. For patients who are ≥80 years of age, hospitalization due to LRTI results in a poor prognosis and a significantly high mortality rate [3]. Therefore, it is important to prevent LRTI-caused readmission and death in octogenarian patients.

Previous studies on factors influencing LRTI-caused readmission and death in the elderly have focused on nutritional status [7, 8], comorbidities [9], gender [2, 10], and swallowing dysfunction [11]. Older adults often suffer from anemia [12, 13]. Most factors that contribute to the development of anemia can be controlled. Hence, etiological treatment may help hinder the progression of anemia. However, anemia in the elderly is often left untreated [13], and previous studies conducted on LRTI-caused readmission have not considered anemia as a risk factor. Furthermore, previous studies on LRTI-caused readmissions have been primarily conducted with patients in the age range of 18–79 years. There is a lack of data on patients older than 80 years.

Therefore, we investigated whether anemia was a risk factor for LRTI-caused readmission and death (occurring within 1 year of discharge from the hospital) in octogenarian patients.

## 2. Methods

### 2.1. Selection and Assignment of Subjects

This was a retrospective cohort study. We used the STROBE cohort reporting guidelines [14]. Patients (age: ≥80 years) hospitalized (in the timespan between 31 March, 2016, and 27 December, 2019) at the Department of Geriatrics, Beijing Tongren Hospital, and suffering from LRTIs were included in the studies. The patients were divided into two groups (anemia and non-anemia) based on the level of hemoglobin (Hb). The patients were followed up for LRTI-caused readmission and death for a period of 1 year after discharge from the hospital. The majority of the patients undergoing follow-up treatment were treated at the same hospital, and data on their readmission and death were obtained from the medical record system of the hospital. In addition, the telephone-based follow-up strategy (the patients (or their family members) were questioned over the telephone) was used to monitor the conditions of the patients.

All patients were divided into two groups (anemia and non-anemia) based on their Hb levels. If the Hb levels were <115 g/L in women or <130 g/L in men, the patient was diagnosed with anemia. The diagnostic criteria were based on the health industry standard for blood cell analysis followed by the People's Republic of China [15]. Patients who died at the time of the first admission or were diagnosed with active malignancy, tuberculosis, gastrointestinal bleeding, achalasia of the cardia, those whose readmission records cannot be queried in the medical record system and that neither patients nor family members can be contacted by telephone, or whose data were missing were excluded from the studies. The patients enrolled in this study all met the following discharge criteria: (1) normal body temperature for more than 5 days, (2) symptoms such as cough, sputum and wheezing were alleviated, and (3) infection indicators such as blood leukocytes and C-reactive protein level were reduced to normal. Therefore, we believe that the hemoglobin level at the time of discharge instead of admission is more indicative of whether the patient has anemia.

### 2.2. Outcomes

Data (date, cause, and the number of times of readmission) on LRTI-caused readmission (within 1 year of discharge) were obtained over a telephonic call and from the medical record system. If the patient died during the period of follow-up, the date and cause of death were recorded. If the patient died during the period of follow-up for reasons other than LRTI, the case was excluded from studies.

### 2.3. Covariates

The following were collected as covariates from the patients: (1) general information (age, gender, and smoking status); (2) information on comorbidities, such as hypertension, ischemic heart disease, diabetes mellitus, chronic heart failure, chronic kidney disease, liver injury, history of cancer, and dementia; (3) details on the history of medication (administration of erythropoietin (EPO) and intake of oral iron, folic acid, oral vitamin B_12_, oral glucocorticoid, inhaled glucocorticoid, statin, and oral amino acid); and (4) laboratory test results (levels of Hb, albumin, total protein, sodium, glucose, high sensitivity C-reactive protein, lipoprotein a, triglyceride, total cholesterol, glycosylated hemoglobin, low-density lipoprotein, and high-density lipoprotein cholesterol). If the patient underwent multiple tests during the period of hospital stay, the results of the tests performed at the time of discharge were used for the studies. The term “biomarkers at discharge” is defined as the last available data on the biomarkers at the time of discharge from hospitals. If multiple data are recorded in a single day, the maximum value was used. Collection and analysis of blood samples were parts of the in-hospital routine.

### 2.4. Statistical Methods

Patients were categorized into anemia and non-anemia groups. Continuous variables such as age and laboratory test results were expressed as mean ± standard deviation, whereas categorical variables such as comorbidities were expressed as percentages. The mean was used as a proxy since a small amount of data was missing (<5%; for glycosylated hemoglobin). For those continuous variables that follow a normal distribution, independent sample *t*-test was conducted, while the rank sum test was conducted for those that follow an abnormal distribution. A chi-square test was conducted to compare differences in categorical variables such as comorbidities. A chi-square test was conducted to assess the differences in the LRTI-caused readmission incidence, the times of readmissions, and the LRTI-related deaths in the two groups. Subsequently, the relative risk corresponding to anemia for LRTI-caused readmission and death and the number needed to treat (NNT) were determined. Finally, a logistic regression model was constructed to analyze the factors associated with LRTI-caused readmission, and a Cox's proportional hazards regression model was constructed to analyze the factors associated with LRTI-caused death. A Kaplan–Meier curve is shown for LRTI-related mortality within 1 year of discharge. The significance level was set at 0.05 (for two-tailed tests). Analyses were performed using IBM SPSS, version 26.

## 3. Results

**Figure 1:**
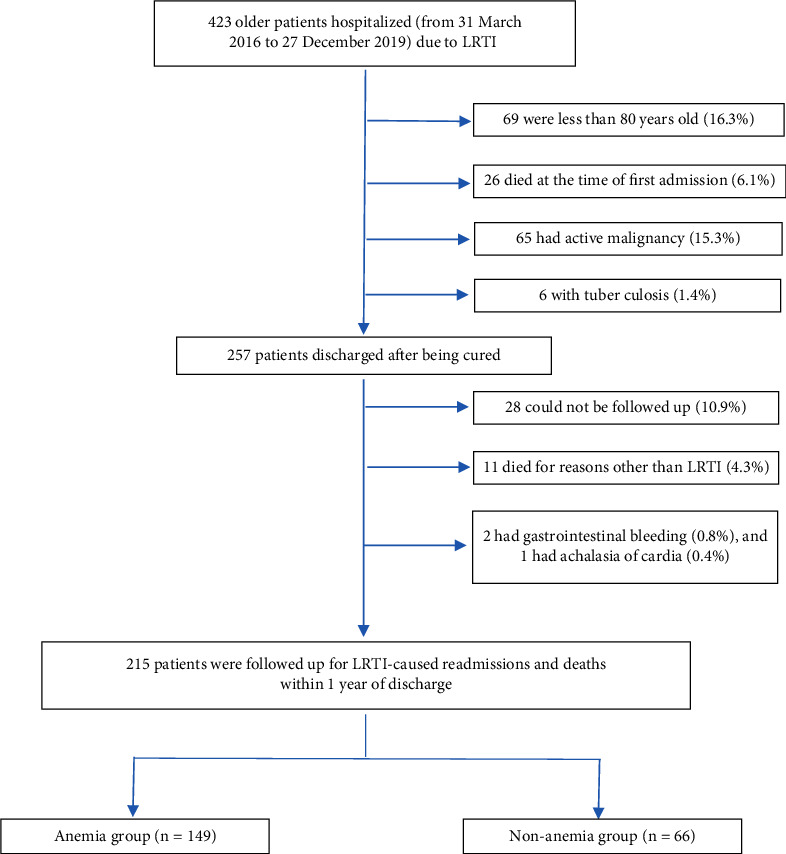
Cohort selection.

A total of 423 patients with LRTI who were hospitalized in the time frame between 31 March, 2016, and 27 December, 2019, were included in this study. Based on the exclusion criteria, 69 patients who were less than 80 years of age (16.3%), 26 patients who died at the time of first admission (6.1%), 65 patients who were diagnosed with active malignancy (15.3%), and 6 patients who were diagnosed with tuberculosis (1.4%) were excluded from the studies. Thus, the conditions of 257 patients discharged from the hospital were followed up for a period of 1 year. Based on the follow-up exclusion criteria, 28 patients whose conditions could not be followed up (10.9%), 11 patients who died for reasons other than LRTI (4.3%), 2 patients with gastrointestinal bleeding (0.8%), and 1 patient exhibiting the conditions of achalasia of cardia (0.4%) were excluded from the studies. Thus, 215 patients (age: >/ = 80 years) who were hospitalized due to LRTI and discharged after being cured were studied. Of these 215 patients, 149 belonged to the anemia group and 66 belonged to the non-anemia group (Figure 1).

The mean age of the cohort at the time of inclusion was 88.73 (SD 4.765) years and 77.2% of the patients were men. The prevalence of anemia was 69.3%.  Table 1 presents the primary characteristics of the study population based on the presence or absence of anemia. There were no differences between the two groups with respect to age, smoking status, comorbidities, the extent of glucocorticoid use (inhaled and oral), and use of lipid-lowering drugs and oral amino. The number of men in the anemia group was higher than that in the non-anemia group. The anemia group was characterized by a higher prevalence of dementia and a poorer nutritional status (as manifested by low total blood protein, albumin, sodium, and lipid levels) compared to the non-anemia group. However, there were no differences in fasting blood glucose and glycosylated hemoglobin levels between the two groups. In the anemia group, 16.8% of patients were treated with EPO and 17.4–22.8% of the patients were treated with oral iron, folic acid, and vitamin B_12_.

The follow-up period was set at 1 year. Overall, 118 patients (i.e., 54.9% of the patients) were readmitted due to LRTI and 48 patients (22.3%) died because of LRTI.  Table 2 presents the incidence rates for readmission and death. The data were obtained based on the degree of anemia. The LRTI-caused readmission observed within 1 year of discharge and the proportion of patients experiencing ≥2 readmissions in the anemia group were significantly higher than the proportion of patients in the non-anemia group (99 (66.4%) vs. 19 (28.8%), *P* < 0.001; 35 (23.5%) vs. 2 (3%), *P* < 0.001). In addition, the incidence rate of LRTI-related death in the anemia group was significantly higher than the rate of the non-anemia group (45 (30.6%) vs. 3 (4.5%), *P* < 0.001).

The risk of LRTI-caused readmission (within 1 year of discharge) in the anemia group was 2.308 times higher than the risk recorded for the non-anemia group. To prevent readmission of one case, 2.65 cases with anemia needed to be treated. Furthermore, the risk of LRTI-related death in the anemia group was 6.644 times higher than the risk recorded for the non-anemia group. To prevent death of one case, 3.9 cases with anemia needed to be treated. According to these results, anemia significantly affected the risk of LRTI-caused readmission and death. Therefore, it is important to treat anemia to prevent LRTI-caused readmission and death.

**Figure 2:**
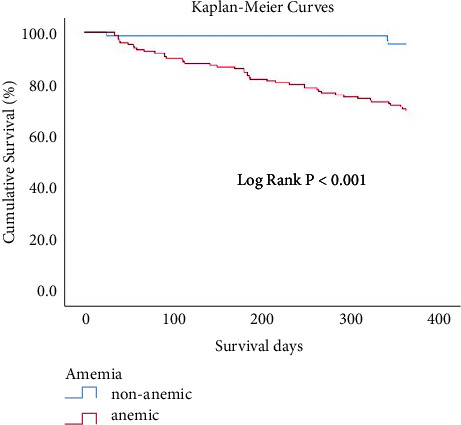
Kaplan–Meier curves for LRTI-related mortality within 1 year of discharge.

Table 3 presents the factors associated with LRTI-caused readmission. Results obtained using the binary logistic regression method revealed that gender (female), hemoglobin level, and albumin level were independent protective factors for readmission attributable to LRTIs (female OR, 0.461; 95% CI, 0.227–0.936; hemoglobin OR, 0.969; 95% CI, 0.950–0.989; albumin OR, 0.861; 95% CI, 0.787–0.942).  Table 4 shows the factors influencing LRTI-related death. Results obtained using the Cox regression method (Enter Method) revealed that the hemoglobin level was also an independent protective factor of death attributable to LRTIs (*β*, −0.037, HR, 0.964; 95% CI, 0.934–0.994). The Kaplan–Meier curve for LRTI-related mortality within 1 year of discharge is shown in Figure 2.

## 4. Discussion

This retrospective cohort study was conducted to examine the effect of anemia on the incidence of LRTI-caused readmission and deaths in octogenarian patients (within 1 year of discharge; mean age: 88.73; SD: 4.765 years). The results revealed a high incidence of anemia (69.3%) in older adults aged ≥80 years who were hospitalized due to LRTI. It was also observed that patients with anemia exhibited a significantly higher incidence rate for LRTI-caused readmission (66.4% vs. 28.8%, *P* < 0.001) and a higher incidence rate for LRTI-caused repeated readmissions (23.5% vs. 3%, *P* < 0.001) than non-anemic patients. The risk of readmission in patients with anemia was 2.308 times higher than the risk suffered by patients belonging to the non-anemia group. Additionally, patients with anemia were more likely to experience LRTI-caused readmission (≥2 times) within 1 year of discharge. To prevent readmission of one case, 2.65 cases with anemia needed to be treated. Further, the risk of LRTI-related death in the anemia group was 6.644 times higher than the risk recorded for the non-anemia group. To prevent the death of one case, 3.9 cases with anemia needed to be treated. The results obtained also suggested that hemoglobin was a protective factor against LRTI-caused readmission and death in the elderly. Therefore, an increase in the Hb levels in the elderly with LRTI can potentially help in reducing the readmission incidence rate and the risk of LRTI-related deaths. However, the proportion of the patients with anemia who were treated with medication was low. Only 16.8% of the patients with anemia included in this study were treated with EPO, and only 17.4–22.8% of the patients with anemia were treated with iron, folic acid, vitamin B_12_, and other hematopoietic ingredients.

LRTI is a prevalent disease that threatens the health of older adults worldwide [2–5]. LRTI increases the medical burden on patients and can also result in functional decline, affecting the activities of daily life [16]. Furthermore, LRTI is the 5th leading cause of death in adults (70 years and older) [6]. For patients ≥80 years old, hospitalization due to LRTI leads to poor prognosis and high mortality rates. Hence, it is important to find ways to prevent the incidence and progression of LRTI in older adults. Previous studies on risk factors associated with LRTI-caused readmission had the following limitations: (1) the duration of follow-up was short, as numerous studies focused on readmission and death within the first 30 days of discharge from the hospital [16–22], (2) the impact of anemia on LRTI-caused readmission and death was not analyzed. The researchers paid more attention to the effects of nutritional status [7, 8], comorbidities [9], gender [2, 10], and swallowing dysfunction [11] on LRTI-caused readmission, (3) previous studies on LRTI-caused readmissions have been primarily conducted with patients in the age range of 18–79 years. There is a lack of data on patients older than 80 years. We investigated the effects of anemia on LRTI-caused readmission and death in octogenarian patients over a one-year follow-up period. In addition, the results reported herein revealed that chronic heart failure and dementia were the risk factors for LRTI-related death. The results also revealed that gender and blood albumin levels were the protective factors for LRTI-caused readmission and death. These results agreed well with previously reported results.

Anemia, as an exposure factor, was chosen for this study because of its high prevalence in older adults. Results from a previous study on the prevalence of anemia in patients from developed countries have revealed that approximately 17% of old persons (>65 years of age) suffered from anemia [12]. A cross-sectional study was conducted in China, and the results revealed that the prevalence of anemia in old people (≥80 years) was 36.0% (95% CI: 30.3–42.0) [13]. The samples used in this study were collected from Beijing, China, so we followed the anemia diagnostic criteria based on the health industry standard for blood cell analysis followed by the People's Republic of China to present the actual picture of the prevalence of anemia in this study sample and its impact on the incidence of LRTI. When the hemoglobin level reaches <115 g/L in women or <130 g/L in men, the patients are diagnosed with anemia. The samples used in previous studies that were conducted on the prevalence of anemia were collected from the community. In contrast, the samples used by us were obtained from patients hospitalized due to LRTI. The prevalence of anemia in the patients considered in this study was significantly higher than the prevalence recorded for the patients considered in previously reported studies. This suggests that there is a correlation between anemia and LRTI.

Based on the etiological classification, anemia can be of various types: nutritional deficiency-based anemia, bleeding anemia, chronic inflammation-based anemia, chronic kidney disease (CKD)-based anemia, and clonal anemia [12]. Often, more than one factor may contribute to the development of anemia in older adults. Lack of iron is the most common cause of anemia, falling under the category of nutritional deficiency-based anemia. Other nutrition-related etiologies include folate and/or Vitamin B_12_ deficiency [18]. The common cause of hemorrhage-induced anemia in older adults can be attributed to the administration of various types of medications (e.g., acetylsalicylic acid and standard (or direct) oral anticoagulants) and gastrointestinal diseases (including cancer) [12]. CKD is another common cause of anemia in older adults. CKD-based anemia primarily results from reduced EPO production and the generation of a blunted response from the erythroid progenitors toward EPO. Other causes of anemia include clonal anemias, such as myelodysplastic syndrome.

Many clinicians consider anemia as a manifestation of aging. Hence, this disorder is often overlooked, resulting in a low treatment rate for anemia in elderly patients. According to the results reported herein, only 16.8–22.8% of the patients with anemia were treated with medication. Therefore, the hemoglobin level in older adults hospitalized due to LRTI should be considered during treatment. If the patients are diagnosed with anemia, the cause should be sought out, and treatment should be provided. A dietician should be appointed for dietary guidance and EPO, folic acid, and vitamin B_12_ should be introduced into the treatment method. Gastrointestinal bleeding should be treated. Regular follow-up visits should be made to monitor the changes in the Hb levels and adjust the treatment plan on time. An improvement in the degree of anemia can help reduce the risk of LRTI-caused readmission and death in older adults, improving their quality of life and reducing the burden on the healthcare system.

### 4.1. Limitations

There are some limitations to this study. First, the sample size of patients without anemia included in this study was small. Second, this retrospective cohort study could not be planned in advance to conduct the laboratory tests. Therefore, data on serum iron, folic acid, vitamin B_12_, EPO, and other indicators associated with anemia were not collected from all the patients. Third, the relatively small sample size of patients in the anemia group did not allow for the grouping of patients into treated and untreated groups to observe the effect of treating anemia on LRTI-caused readmission. Finally, given the variation in normal hemoglobin levels between different regions and ethnicities, the diagnostic criteria for anemia were based on the health industry standard for blood cell analysis followed by the People's Republic of China. The diagnostic criteria outlined by WHO were not taken into account.

## 5. Conclusions

LRTI in octogenarian patients is a highly prevalent disorder associated with a poor quality of life, worse outcomes, and increased mortality rates. Anemia is a risk factor for LRTI-caused readmission and death in octogenarian patients. It is often overlooked and left untreated. LRTI-caused readmission and death within 1 year of discharge from the hospital can be reduced by improving the degree of anemia or reducing the incidence rate of anemia. Anemia in older adults is often the consequence of a combination of etiologies. Therefore, clinicians should actively provide treatment to reduce the incidence rate or degree of anemia to reduce the risk of LRTI-caused readmission and deaths and improve the quality of life of elderly patients.

## Figures and Tables

**Table 1:** Baseline characteristics of the study population.

Characteristics	Anemia (*n* = 149)	No anemia (*n* = 66)	*P* value
Age, mean (SD), y	89.12 (4.9)	87.86 (4.3)	0.070
Male, no. (%)	127 (85.2)	39 (59.1)	<0.001
Smoking, no. (%)	5 (3.4)	7 (10.6)	0.070

*Comorbidity, no. (%)*			
Hypertension	111 (74.5)	53 (80.3)	0.36
Ischemic heart disease	83 (55.7)	29 (43.9)	0.11
Chronic heart failure (NYHA)			
Class I-II, no. (%)	113 (75.8)	52 (78.8)	0.64
Diabetes mellitus	69 (46.3)	31 (47)	0.93
Chronic kidney disease			
≥3 stage	53 (35.6)	23 (34.8)	0.92
Liver injury	17 (11.4)	6 (9.1)	0.61
History of cancer	24 (16.1)	8 (12.1)	0.45
Dementia	52 (34.9)	10 (15.2)	0.003

*Medicine, no. (%)*			
Erythropoietin	25 (16.8)	1 (1.5)	0.002
Oral iron	34 (22.8)	2 (3.0)	<0.001
Folic acid	26 (17.4)	1 (1.5)	0.001
Vitamin B_12_	30 (20.1)	6 (9.1)	0.045
Oral glucocorticoid	11 (7.4)	5 (7.6)	1.00
Inhaled glucocorticoid	11 (7.4)	6 (9.1)	0.67
Statin	80 (53.7)	39 (59.1)	0.46
Oral amino acid	19 (13.0)	4 (6.3)	0.14

*Laboratory test, mean (SD)*			
Hemoglobin, mean (SD), g/L	105.63 (12.88)	132.32 (9.23)	<0.001
Total protein, mean (SD), g/L	60.67 (7.97)	63.46 (5.87)	<0.001
Albumin, mean (SD), g/L	31.26 (3.60)	35.12 (3.57)	<0.001
Sodium, mean (SD), mmol/L	136.8 (10.95)	139.7 (3.80)	0.002
Glucose, mean (SD), mmol/L	6.18 (2.16)	5.80 (1.68)	0.65
Total cholesterol, mean (SD), mmol/L	3.46 (0.77)	4.05 (0.90)	<0.001
Triglyceride, mean (SD), mmol/L	0.93 (0.56)	1.01 (0.52)	0.013
Low-density lipoprotein cholesterol, mean (SD), mmol/L	1.91 (0.78)	2.33 (0.75)	<0.001
High-density lipoprotein cholesterol, mean (SD), mmol/L	1.02 (0.35)	1.13 (0.35)	0.031
Glycosylated hemoglobin (HBA1c), mean (SD), %	6.37 (0.97)	6.48 (1.14)	0.98

SD, standard deviation; NYHA, New York heart association.

**Table 2:** Readmission and death within 1 year of discharge: Analysis of the study population.

Outcomes	Anemia (*n* = 149)	No anemia (*n* = 66)	*P* value
Readmission, no. (%)	99 (66.4)	19 (28.8)	<0.001
Death, no. (%)	45 (30.6)	3 (4.5)	<0.001
*Number of times of readmission*			
Once, no. (%)	64 (43)	17 (25.8)	<0.001
≥2 times (%)	35 (23.5)	2 (3.0)	<0.001

**Table 3:** Logistic regression analysis: Related factors of readmission attributable to LRTIs within 1 year of discharge.

Factor	OR (95% CI)	*P* value
*Univariate*		
Sex, female	0.429 (0.223–0.823)	0.011
Hemoglobin	0.956 (0.938–0.974)	<0.001
Albumin	0.809 (0.745–0.878)	<0.001
Total cholesterol	0.613 (0.440–0.853)	0.004
Low-density lipoprotein cholesterol	0.693 (0.481–1.000)	0.050
High-density lipoprotein cholesterol	0.455 (0.208–0.995)	0.049

*Multivariate*		
Sex, female	0.461 (0.227–0.936)	0.032
Hemoglobin	0.969 (0.950–0.989)	0.003
Albumin	0.861 (0.787–0.942)	0.001

Sex, male = 1, female = 2. OR, odds ratio, CI, confidence interval.

**Table 4:** Cox's proportional hazards regression model (enter method): Related factors of death related to LRTIs within 1 year of discharge.

Factor	*β*	HR (95% CI)	*P* value
Hemoglobin	−0.037	0.964 (0.934–0.994)	0.019
Dementia	−1.232	0.292 (0.129–0.661)	0.003
Age	0.071	1.073 (0.989–1.165)	0.090

HR: hazard ratio, CI: confidence interval.

## Data Availability

All data relevant to the study are available on reasonable request to the corresponding author.

## Supplementary Materials

Supplementary Materials For the analysis of risk factors for death within one year of discharge of lower respiratory tract infection, we have used the Cox proportional hazards regression model. In the manuscript we showed the statistic results of the “Enter” method, while we also conducted several other different statistic methods such as Forward: Conditional, Forward: LR, Forward: Wald, Backward: Conditional, Backward: LR, Backward: Wald. We listed all of the results in our supplement file for information. Of all the results of different methods, hemoglobin is statistically significant.
