# Supplementary material for: Effect of Anemia on Readmission and Death in Octogenarian Patients with Lower Respiratory Tract Infections: A Retrospective Cohort Study
Source: Int J Clin Pract. 2022 Oct 27;2022:4566936. doi: 10.1155/2022/4566936 (PMC9633185; doi:10.1155/2022/4566936)
Supplement: Supplementary Materials — For the analysis of risk factors for death within one year of discharge of lower respiratory tract infection, we have used the Cox proportional hazards regression model. In the manuscript we showed the statistic results of the “Enter” method, while we also conducted several other different statistic methods such as Forward: Conditional, Forward: LR, Forward: Wald, Backward: Conditional, Backward: LR, Backward: Wald. We listed all of the results in our supplement file for information. Of all the results of different methods, hemoglobin is statistically significant. [file 4566936.f1.docx]

| Cox’s Proportional Hazards Regression Model (**Forward: Conditional**): Related Factors of Death Related to LRTIs Within 1 Year of Discharge | | | | | | |
| --- | --- | --- | --- | --- | --- | --- |
| Factor | β | HR (95% CI) | | *P* value | | |
| Hemoglobin | -0.025 | 0.975 (0.954-0.997) | | 0.025 | | |
| Albumin | -0.101 | 0.904 (0.826-0.989) | | 0.028 | | |
| Creatinine | 0.003 | 1.003 (1.001-1.005) | | 0.008 | | |
|  |  |  |  |  |  |  |
|  |  |  |  |  |  |  |
| Cox’s Proportional Hazards Regression Model (**Forward: LR**): Related Factors of Death Related to LRTIs Within 1 Year of Discharge | | | | | | |
| Factor | β | HR (95% CI) | | *P* value | | |
| Hemoglobin | -0.025 | 0.975 (0.954-0.997) | | 0.025 | | |
| Albumin | -0.101 | 0.904 (0.826-0.989) | | 0.028 | | |
| Creatinine | 0.003 | 1.003 (1.001-1.005) | | 0.008 | | |
|  |  |  |  |  |  |  |
|  |  |  |  |  |  |  |
| Cox’s Proportional Hazards Regression Model (**Forward: Wald**): Related Factors of Death Related to LRTIs Within 1 Year of Discharge | | | | | | |
| Factor | β | HR (95% CI) | | *P* value | | |
| Hemoglobin | -0.025 | 0.975 (0.954-0.997) | | 0.025 | | |
| Albumin | -0.101 | 0.904 (0.826-0.989) | | 0.028 | | |
| Creatinine | 0.003 | 1.003 (1.001-1.005) | | 0.008 | | |
|  |  |  |  |  |  |  |
|  |  |  |  |  |  |  |
| Cox’s Proportional Hazards Regression Model (**Backward: Conditional**): Related Factors of Death Related to LRTIs Within 1 Year of Discharge | | | | | | |
| Factor | β | HR (95% CI) | | *P* value | | |
| Hemoglobin | -0.047 | 0.954 (0.933-0.976) | | ＜0.001 | | |
| Creatinine | 0.003 | 1.003 (1.000-1.006) | | 0.027 | | |
|  |  |  |  |  |  |  |
| Cox’s Proportional Hazards Regression Model (**Backward: LR**): Related Factors of Death Related to LRTIs Within 1 Year of Discharge | | | | | | |
| Factor | β | HR (95% CI) | | *P* value | | |
| Hemoglobin | -0.047 | 0.954 (0.933-0.976) | | ＜0.001 | | |
| Creatinine | 0.003 | 1.003 (1.000-1.006) | | 0.027 | | |
|  |  |  |  |  |  |  |
|  |  |  |  |  |  |  |
| Cox’s Proportional Hazards Regression Model (**Backward: Wald**): Related Factors of Death Related to LRTIs Within 1 Year of Discharge | | | | | | |
| Factor | β | HR (95% CI) | | *P* value | | |
| Hemoglobin | -0.032 | 0.968 (0.946-0.992) | | 0.008 | | |
| Creatinine | 0.004 | 1.004 (1.001-1.006) | | 0.003 | | |
|  |  |  |  |  |  |  |
